# Natural Language Processing–Based Virtual Cofacilitator for Online Cancer Support Groups: Protocol for an Algorithm Development and Validation Study

**DOI:** 10.2196/21453

**Published:** 2021-01-07

**Authors:** Yvonne W Leung, Elise Wouterloot, Achini Adikari, Graeme Hirst, Daswin de Silva, Jiahui Wong, Jacqueline L Bender, Mathew Gancarz, David Gratzer, Damminda Alahakoon, Mary Jane Esplen

**Affiliations:** 1 de Souza Institute University Health Network Toronto, ON Canada; 2 Department of Psychiatry Faculty of Medicine University of Toronto Toronto, ON Canada; 3 Princess Margaret Cancer Centre University Health Network Toronto, ON Canada; 4 Centre for Data Analytics and Cognition La Trobe University Melbourne Australia; 5 Department of Computer Science University of Toronto Toronto, ON Canada; 6 Dalla Lana School of Public Health University of Toronto Toronto, ON Canada; 7 Centre for Addiction and Mental Health Toronto, ON Canada

**Keywords:** artificial intelligence, cancer, online support groups, emotional distress, natural language processing, participant engagement

## Abstract

**Background:**

Cancer and its treatment can significantly impact the short- and long-term psychological well-being of patients and families. Emotional distress and depressive symptomatology are often associated with poor treatment adherence, reduced quality of life, and higher mortality. Cancer support groups, especially those led by health care professionals, provide a safe place for participants to discuss fear, normalize stress reactions, share solidarity, and learn about effective strategies to build resilience and enhance coping. However, in-person support groups may not always be accessible to individuals; geographic distance is one of the barriers for access, and compromised physical condition (eg, fatigue, pain) is another. Emerging evidence supports the effectiveness of online support groups in reducing access barriers. Text-based and professional-led online support groups have been offered by Cancer Chat Canada. Participants join the group discussion using text in real time. However, therapist leaders report some challenges leading text-based online support groups in the absence of visual cues, particularly in tracking participant distress. With multiple participants typing at the same time, the nuances of the text messages or red flags for distress can sometimes be missed. Recent advances in artificial intelligence such as deep learning–based natural language processing offer potential solutions. This technology can be used to analyze online support group text data to track participants’ expressed emotional distress, including fear, sadness, and hopelessness. Artificial intelligence allows session activities to be monitored in real time and alerts the therapist to participant disengagement.

**Objective:**

We aim to develop and evaluate an artificial intelligence–based cofacilitator prototype to track and monitor online support group participants’ distress through real-time analysis of text-based messages posted during synchronous sessions.

**Methods:**

An artificial intelligence–based cofacilitator will be developed to identify participants who are at-risk for increased emotional distress and track participant engagement and in-session group cohesion levels, providing real-time alerts for therapist to follow-up; generate postsession participant profiles that contain discussion content keywords and emotion profiles for each session; and automatically suggest tailored resources to participants according to their needs. The study is designed to be conducted in 4 phases consisting of (1) development based on a subset of data and an existing natural language processing framework, (2) performance evaluation using human scoring, (3) beta testing, and (4) user experience evaluation.

**Results:**

This study received ethics approval in August 2019. Phase 1, development of an artificial intelligence–based cofacilitator, was completed in January 2020. As of December 2020, phase 2 is underway. The study is expected to be completed by September 2021.

**Conclusions:**

An artificial intelligence–based cofacilitator offers a promising new mode of delivery of person-centered online support groups tailored to individual needs.

**International Registered Report Identifier (IRRID):**

DERR1-10.2196/21453

## Introduction

### Background

A cancer diagnosis, and its subsequent treatment, has a significant impact on short- and long-term psychological well-being. Many patients and caregivers experience a range of distressing symptoms including worry, fear, irritability, sleeplessness, and guilt, while others develop more severe symptoms such as loss of interest in life [1-3]. Approximately half of all individuals in cancer treatment report undue and persistent emotional distress [4-7]. Persistent distress is associated with poor treatment adherence [8], reduced health-related quality of life [9], and higher mortality [10].

Online support groups are effective in reducing emotional distress [11-13]. They are a convenient alternative for those who cannot attend in-person support groups because of geographic distance, physical health concerns, and other responsibilities [14]. While there are a variety of delivery methods (eg, video or text), professionally led online support groups are typically synchronous (in real time) where participants engage in therapeutic interactions with a therapist and other participants in the group. Group interactions can be recorded and reviewed by participants during or postsession. Cancer Chat Canada (CCC) offers professionally led text-based support groups to patients with cancer and caregivers across Canada, with transcripts available to therapists and participants for postsession review. The online support group in CCC is manual-based, with session-specific themes, readings, and interactive activities. Therapists lead group discussions based on the manual, address new topics brought up by participants during the session, attend to an individual's emotional concerns, and facilitate mutual support.

The helpful therapeutic aspects of online support groups include facilitation of information sharing, expression of emotions, and provision of support as well as coping strategies [13,15]. Participants quickly become accustomed to text-based interactions and show increased feelings of empowerment, reduced social isolation, better managed stress, and improved coping [13]. Emotional expression is expected and encouraged by the therapists, particularly the emotions that participants may consider negative such as sadness, anger, or fear [3]. However, therapists need to gauge the frequency and intensity of expressed emotions from participants and address them in a timely fashion. In text-based online support groups, therapists do not have visual cues and may miss participants’ emotional needs when entries by multiple participants are posted simultaneously on a fast-scrolling screen [16,17]. Failure to recognize and respond to emotional distress expressed by participants can have clinical consequences, such as reduced perceived support and increased group dropouts. These challenges can also make a well-trained therapist feel frustrated and limit their uptake of this form of support group delivery. Another unique challenge is participant engagement; online support group therapists find it difficult to foster group cohesion. They find it harder to promote belonging and deepen group members’ connections with one another without visual cues. Both engagement and group cohesion are imperative for optimal outcomes [18,19].

Currently, little is known concerning the mechanisms related to the process of online delivery that can support therapists in delivering optimal virtual online group support. To date, few studies have focused on examining mechanisms of action in therapist-led online support group, such as active therapeutic ingredients, mediators of change, or participant distress which may be associated with reduced level of engagement and participant dropout rates [18]. With the increased popularity of virtual care, more research is needed to understand what factors enhance (or impede) specific patient outcomes for evidence-based programs.

Artificial intelligence (AI) methods, such as deep-learning based natural language processing (NLP), offer a novel solution to addressing these challenges by capturing participants’ emotional expression (including distress), level of engagement, and group cohesion in real time during text-based sessions, without imposing a measurement burden [20,21]. For example, if methods of deep learning–based NLP can systematically identify important emotions, such as fear, anxiety, sadness, and depression, then assistance in signaling and monitoring participants who need additional support can be provided. Tracking and monitoring participants’ cognitive-emotional states using AI approaches may reduce attrition, improve therapeutic outcomes, and allow therapists to focus their attention more fully on the therapeutic encounters [22].

We aim to explore whether deep learning–based NLP can optimize online support group delivery through the creation of an artificial intelligence–based cofacilitator (AICF). AICF training, testing, implementation, and evaluation uses textual data from the CCC therapist-led online support group service. The AICF prototype will be adapted from an AI-based framework called Patient-Reported Information Multidimensional Exploration (PRIME) for detection of emotional states, piloted on data from patients with prostate cancer in Australia [23-25].

### The PRIME Framework

This study is built upon the PRIME framework. De Silva et al [24] pioneered AI research for automatic identification of physical and emotional states of patients with prostate cancer for active surveillance to identify their psychosocial needs. The PRIME framework, an automated ensemble of deep-learning based NLP techniques, was developed to analyze text from online patient forums. PRIME has been used to analyze 10 high-volume online patient forums consisting of 22,233 patients with prostate cancer, which generated a text data set of 609,960 conversations. PRIME demonstrated its capabilities in identifying diverse physical symptoms, and functional and emotional outcomes (eg, sadness, anger, confusion) embedded in forum discussions. It generates visualizations of aggregated expressions of emotion trajectories by patient-reported demographics, decisions, treatment, and side effects [23-25].

**Figure 1:**
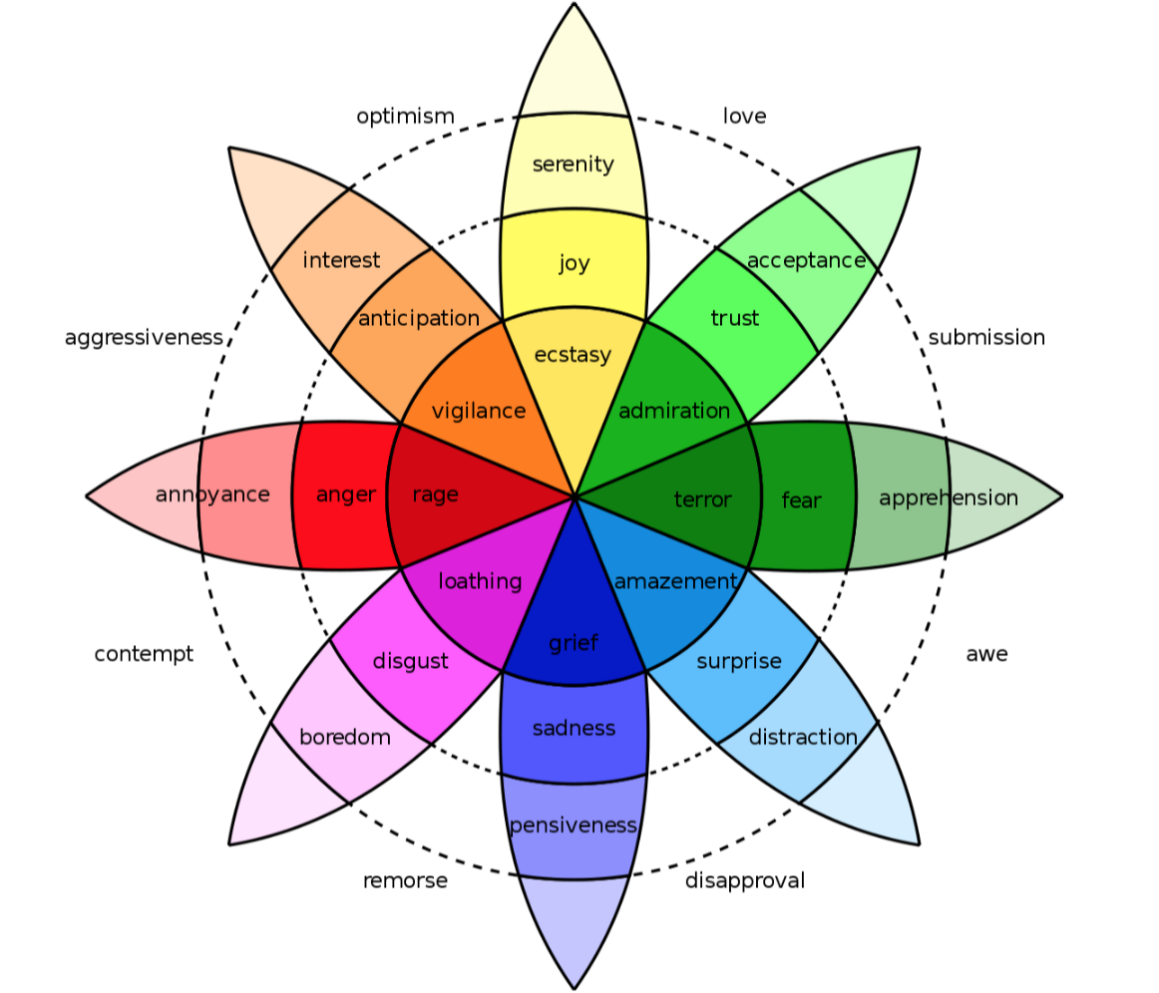
Plutchik [31] wheel of basic emotions, basis for the PRIME emotion categories.

PRIME extracts emotions comprehensively by parsing a body of text to different levels of granularity: word, phrase, sentence, post, and all posts of each user, using multiple techniques (vector space modeling, topic modeling, word2vec [26,27], SentiWordNet [28], and named entity recognition) and SNOMED terms [29]. These techniques are used to identify a group of key words associated with a topic as a context variable based on an aggregated measure of features derived at each level, and in turn, determine eight basic emotion categories [30,31], their strengths, and overlaps (eg, anxiety is an overlap between fear and sadness) [24]. The basic emotion categories are based on the Plutchik [31] wheel of basic emotions, illustrated in Figure 1. Each level of extraction will serve as an input for calibration of the subsequent extraction to increase accuracy [23-25]. PRIME will serve as a foundation for the current AICF project algorithm, as outlined below.

An AICF will track and monitor online support group participants’ emotional distress through real-time analysis of text-based messages posted during sessions. Specifically, the AICF aims to (1) identify participants who are disengaged or at-risk for increased emotional distress and track in-session engagement and group cohesion levels, providing real-time alerts for therapist follow-up; (2) generate postsession participant profiles that contain discussion content keywords and emotion profiles for each session; and (3) automatically suggest tailored resources to participants according to their needs.

## Methods

### Overview

This project has 4 phases: (1) developing an AICF using a subset of existing CCC data based on PRIME, (2) evaluating its performance using human scoring, (3) beta testing the AICF within CCC, and (4) evaluating user experiences. This study will be conducted in compliance with the principles of the Declaration of Helsinki.

### Phase 1: Developing an AICF

#### Summary

**Figure 2:**
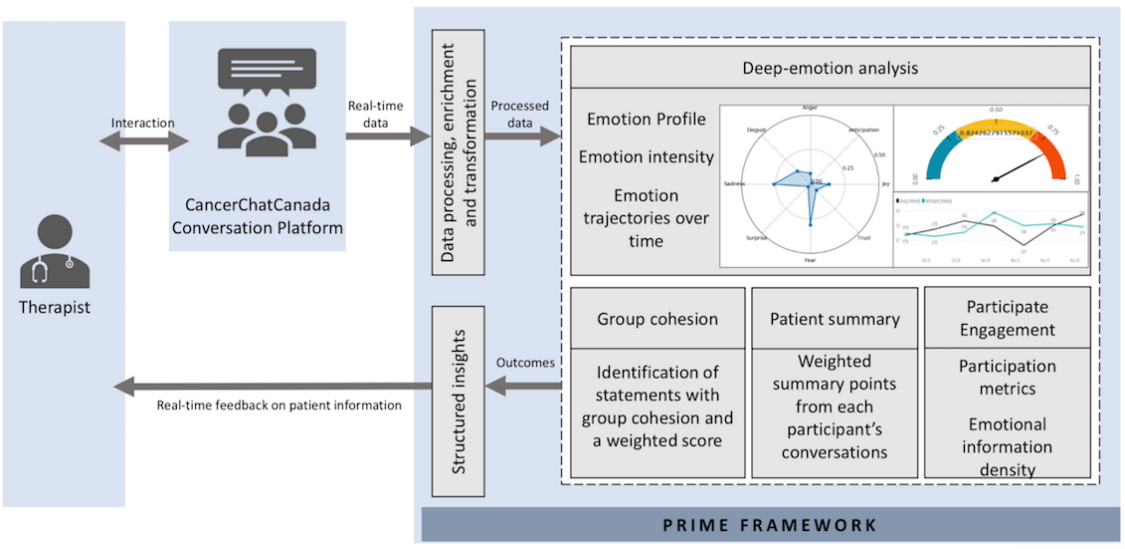
Functionalities of AICF. PRIME: Patient-Reported Information Multidimensional Exploration.

In addition to the 8 basic emotions detected by PRIME, the AICF will include additional functionality to support (1) real-time monitoring and alerting of emotional distress, participant engagement, and group cohesion, (2) participant emotional profiling, and (3) tailored resource recommendations. Figure 2 describes the functionalities and process of transforming PRIME to an AICF.

To inform development of the AICF, CCC data from 430 unique participants (approximately 80,000 conversations) across multiple sessions in 2 years will be used. The majority of participants were females, aged between 45 and 64 years, living in suburban or rural areas of British Columbia or Ontario. Many had breast cancer and were in the posttreatment period. See participant characteristics in Table 1.

The text data were deidentified to ensure confidentiality, using a risk-based approach in 2 steps. The first step used an open-source clinical text deidentification library (philter-ucsf [32]) to replace identifiers such as name, address, sex, age or year mention, and health care organization (hospital) names with asterisks, preserving word length. The second step involved human review and deidentification of any identifiers that the library missed. The AICF was trained using the deidentified data.

**Table 1:** CCC participant characteristics.

Characteristics		Value (n=430), n (%)
**Gender**		
	Female	383 (89.0)
	Male	47 (11.0)
**Age group (years)**		
	18-24	6 (1.5)
	25-34	27 (6.2)
	35-44	66 (15.3)
	45-54	130 (30.2)
	55-64	147 (34.2)
	65+	54 (12.6)
**Location**		
	British Columbia	146 (34.0)
	Ontario	142 (33.0)
	Alberta	52 (12.0)
	Other province	90 (21.0)
**Geography**		
	Urban	168 (39.0)
	Suburban/rural area	262 (61.0)
**Type of cancer**		
	Breast	133 (31.0)
	Gynecological	90 (21.0)
	Colorectal	43 (10.0)
	Head and neck	13 (3.0)
	Other cancers	151 (35.0)
**Treatment status**		
	Active treatment	116 (27.0)
	Posttreatment	176 (41.0)
	Unknown	138 (32.0)

#### Functionality 1: Real-Time Monitoring and Alert

The AICF is designed to track emotional distress, participant engagement, and group cohesion. First, conversational extracts and user behaviors (eg, emotional intensity, participant engagement) will be used to train an ensemble of machine learning algorithms [33] that predict the likelihood of significant emotional distress. This ensemble method generates a weighted score that offsets potential bias in the 2 approaches as well as takes both emotion and engagement into account. Manually annotated texts of instances of distress were used to train the AICF. For the purpose of assessing the intensity of distress, 10 sessions of chat data were annotated and classified according to levels of distress (none, low, medium, or high) in the participants' statements. Based on the focus group feedback, the therapists highlighted that, apart from the 8 basic emotions, it is important to specifically detect participants' comments indicating hopelessness, distress, and loneliness, which are more complex emotional experiences specific to a cancer population [34,35]. Identifying these emotions are key in cancer online support groups as they are linked to depression (hopelessness, loneliness) [4] and poor outcomes (distress) [8]. Therefore, emotion intensities for all 11 emotions have been aggregated into group- and participant-specific emotion intensities. The AICF will produce risk scores of significant distress and that will be displayed and updated at each 30-minute interval on a 90-minute session timeline. If a participant’s risk score has increased significantly compared to the previous intervals, then the therapist will be alerted. The alert threshold will be finalized in the user testing phase.

Participant engagement is defined by the information density of their text posts, which consists of the emotional content detected by the AICF, number of words per post, and frequency of posting. The AICF will update every 5 minutes to show if participants are participating at high, medium, or low levels or if they have not posted in some time. Real-time monitoring of participant engagement, relative to the group will also be displayed on the real-time dashboard.

Group cohesion is defined as a sense of belonging to and feeling supported by the group [36]. This is indicated by referring to other participants as “us,” “we,” and “our group”; expressing gratitude for sharing or help from others; commenting that they are looking forward to the next group; or chatting together outside of group time. The AICF will capture the group cohesive statements and present an aggregated score that denotes the level of group cohesiveness among all members. Group cohesive statements (100 examples) were annotated by the therapists from the CCC data, and keywords and phrases derived from these examples were used as seed terms in a word embedding model.

#### Functionality 2: Participant Emotional Profiling

The AICF will use features from PRIME to generate a collection of key phrases and topics and an emotional profile for each participant over a 90-minute session. The summary will consist of participant clinical and sociodemographic characteristics and will display key phrases that indicate emotional distress and the intensity [23] of emotion associated with each concern on a 90-minute timeline. The display time window can be expanded as sessions continue.

#### Functionality 3: Tailored Resource Recommendations

The AICF will use outcomes from the first 2 functionalities to train an unsupervised incremental learning algorithm that merges similar participant profiles and differentiates dissimilar ones. The incremental learning feature will maintain these learned groupings (eg, presurgery vs postsurgery) over time, uncovering shared group behaviors and patterns. These will then be combined with known participant characteristics (eg, location, age), use of psychosocial resources (eg, sexual health clinic), and timing for a specific issue (eg, sexual health) from CCC data as inputs to a recommender system. Using association rule mining algorithms, a list of resources with predicted scores will be generated; resources with the highest predicted scores will be recommended to a participant [37]. Test data will be used to evaluate relevance and reliability of each recommendation. This functionality will increase tangible support and service quality without incurring increased workload on the therapists.

### Phase 2: Human Scoring

To develop the AICF, the emotion labeling outputs will be scored by a team of psychology undergraduate and graduate students, including 2 doctoral-level clinical psychology therapists. The team will score 20% of the output as feedback to retrain the AICF. This updated version will be run on the remaining 80% of the data. Each AICF version will be saved before it is trained on new data. To evaluate the AICF, 2 online support group groups’ messages will be withheld for the final testing phase of the AICF.

The team will score the output using guides based on definitions from the literature and examples from well-established psychometric measures (Table 2). For example, sadness and fear were selected for the first round of human scoring due to their relevance as symptoms of anxiety and depression, and they were the emotions in the high distress post category most commonly identified by annotators. The output will be scored at the sentence level, such that the target output must be clearly present in the sentence, without additional textual context.

**Table 2:** Target measures for human scoring.

Output	Category	Description
Sadness	Emotion	Expressing loss, grief, unhappiness, hopelessness; feeling low, down, or blue; behaviors such as crying, withdrawing. Possible indicator of depression (persistent, high distress [31])
Fear	Emotion	Feeling scared, panicked, alarmed, apprehensive; less intensely worried, anxious, stressed, irritable, tense; being unable to focus, relax. Possible indicator of anxiety (persistent, high distress [31])
Group cohesion	Group process	A culmination of participation, engagement, expressed mutual support, gratitude for other members, looking forward to future chat sessions and referring to the group as “we” and “us” to indicate a sense of belonging [38].
Emotional profiling	Postgroup feature	A text summary of key phrases with high emotional content for each participant, scored for accuracy.
Tailored resource recommendation	Postgroup feature	A list of relevant psychosocial resources will be generated for each participant based on the conversations during the session.

Using the literature, the scorers will note the instances in which the AICF has (1) correctly identified each output instance (true positive); (2) incorrectly identified an output instance (false positive); (3) correctly identified the lack of an output (true negative); or (4) missed an output in a sentence (false negative). For example, from the sentence “Yesterday I had a melt down, just felt so sad and cried, it came out of nowhere,” the AICF correctly identified the comment's sentiment as sadness and so did the human scorer (true positive). From the sentence “I can still laugh at some pretty bad jokes,” the AICF incorrectly identified the comment's sentiment as sadness, whereas the human scorer rated the comment's sentiments as no sadness (false positive). To judge the emotional intensities generated by the AICF, the scorers defined 4 levels of distress: low, moderate, moderate-high, and high.

Upon completion of the scoring, AICF performance will be evaluated for sadness, fear, and group cohesion measures using recall, precision, and F1 score. Precision is defined as a measure of result relevancy while recall is defined as a measure of how many truly relevant results are captured. The F1 score [39], which is the weighted average of precision and recall, takes both false positives and false negatives into account. For F1 scores below 80%, scorer feedback will be used to improve the AICF until it achieves 80%. The scoring results will be used to generate the list of keywords of queried expressions in the word embedding model, while linguistic rules will be added to handle exceptions such as negations, idioms, irony, or expressed sarcasms that are unique to participants with cancer. This feedback loop will improve the performance of each functionality using domain expertise [40] to produce an acceptable evaluation F1 score.

### Phase 3: Beta Testing

#### Summary

The AICF will be deployed and tested in the CCC platform background (out of the therapists’ view) in 3 groups. It will then be run for therapist use and feedback for beta testing on 10-12 groups to analyze the performance of the AICF system output, such as all emotions expressed (including distress), intensity, group cohesion, engagement, and emotional profiling features (see Table 2). We hypothesize that the AICF output will be highly correlated with standard clinical measures of psychological outcomes and have high sensitivity and predictive values for distress. These quantitative evaluations will provide evidence to support the AICF’s validity and reliability.

#### Design

A single-arm trial to evaluate the AICF’s validity and reliability among CCC therapists and participants.

#### Participants

Ten therapists and 100 support group participants (ie, patients and caregivers) (10-12 groups) will be recruited through a multipronged approach, including in-person (University Health Network clinics), print (flyers and posters posted at University Health Network locations), and digital media (eg, Twitter, Google, and Facebook [41], CCC platform, and webpages of CCC provincial partners across Canada). In-person recruitment will take place at University Health Network clinics using protocols approved by the University Health Network research ethics board. A study coordinator will explain the study prior to informed consent. For online and print recruitment, respondents will be provided with the study webpage and phone number of the research team for study inquiry. Interested patients will be followed-up by a call from the study coordinator for study details. Study log will be maintained and reasons for nonparticipation collected. The existing CCC therapist roster will be used to recruit therapist-participants directly. Therapists will receive training on the AICF. An estimated 120,000 posts (200 posts/user/session × 6 sessions × 100 users) will be generated, sufficient for the sensitivity and specificity analysis [42]. This study has been approved by the University Health Network research ethics board.

#### Measures

Participant distress will be assessed by standardized measures pre and postprogram by several scales. The Impact of Event Scale-Revised [43] is composed of 22 items rated on a 5-point Likert scale, yielding a total score ranging from 0 to 88—8 items on intrusion (Cronbach α=.87-.94), 8 items on avoidance (Cronbach α=.84-.87), and 6 items on hyperarousal (Cronbach α=.79-.91). The 7-item Hospital Anxiety and Depression Scale (HADS) [44], with items rated on a scale of 0 to 3, yielding a total score ranging from 0 to 21 has 2 subscales: anxiety and depression (Cronbach α=.88 and .92, respectively) [44]. The 18-item Brief Symptom Inventory [45] is rated on a 5-point Likert scale, and the sum is transformed into a T-score. The Brief Symptom Inventory is composed of 3 dimensions: somatization, depression, and anxiety (Cronbach α=.71-.85) [45]. A participant will be defined as having significant distress if they score above the cut-offs for any of the scales: cancer-related distress (Impact of Event Scale-Revised score >24), symptoms of depression (HADS score>10), or anxiety (Brief Symptom Inventory T-score >60).

Participant postsession emotionality will be assessed using the Positive and Negative Affect Schedule [46], a 20-item self-report measure of positive and negative affect (Cronbach α=.89 and .85, respectively). Postprogram group cohesion will be measured using the 19-item Therapeutic Factor Inventory [47]—Instillation of Hope (4 items), Secure Emotional Expression (7 items), Awareness of Interpersonal Impact (5 items), and Social Learning (3 items). The items are rated on a scale from 1 to 7 and a mean score is based on a sum of the item ratings multiplied by factor score weightings (Cronbach α=.71-.91) [47]. Therapists will assess the AICF’s usability postsession using the 10-item System Usability Scale using a 5-point Likert scale of agreement with scores ranging from 0 to 100 (Cronbach α=.95)[48]. Finally, the online support group experience will be measured using the 24-item Counsellor Activity Self-Efficacy Scale [49], with items rated on a 10-point Likert scale (Cronbach α=.96) [49].

#### Emoji Scale

Emoji scales validated as representations of physical and emotional quality of life in cancer populations [50] will be used to track emotions of the participants during each session. For AICF validation, we will employ an automatic check-in that will occur at three 30-minute intervals using 9 different emoticons (eg, worried, sad, supported) from which participants can choose to represent their emotional states in the moment. Each participant will provide up to 300 emoji ratings (3×10 sessions).

#### Statistical Analysis

The ability of the AICF to correctly identify distress will be assessed. First, a chi-square test will be used to assess the sensitivity and specificity of the AICF against the self-reported emoji at the 30-minute interval. Second, linguistics inquiry word count [51] will be performed on each post. Linguistics inquiry word count scans each post for the linguistic markers of distress (eg, first-person singular pronouns, and words that Linguistics inquiry word count classifies as sad, anxiety or fillers) and provides a correlation coefficient. We hypothesize that correlations between linguistics inquiry word count and AICF output would be strong (≥0.7). Third, a precision-recall curve (positive predictive value vs sensitivity) will be used to map AICF classifications against established cut-offs of a standardized measure. An area under the curve >80% would be considered high performance [52]. The precision-recall curve can be used to inform the statistical threshold for distress that warrants a therapist alert. Fourth, construct (convergent) validity of AICF sentiment analysis will be compared against self-reported standardized measures. We hypothesize that all positive and negative emotions extracted will be strongly correlated (Pearson correlation coefficient ≥0.7) with Positive and Negative Affect Schedule subscales scores. For example, the extracted negative emotions will be positively correlated with HADS. Finally, internal consistency will be measured among extracted negative and positive emotions using Cronbach α.

### Phase 4: Evaluation of User Experiences

Participants will rate their satisfaction with their online support group experience (eg, group cohesion) using the Therapeutic Factor Inventory. Therapists will provide ratings of system usability (with the System Usability Scale) and perceived self-efficacy measure (with the Counsellor Activity Self-Efficacy Scale) in leading the groups. All of these ratings are expected to be high (>80th percentile). Quality indicators, such as study attendance and dropout rate (defined as participants missing more than 2/10 sessions) will be compared using chi-square tests to those in a special topic group (eg, sexual health group). We expect that the dropout rate will be 50% less than those of existing groups.

## Results

Phase 1 was initiated in August 2019 and completed in December 2019. Phase 2 was initiated in January 2020, and as of December 2020, is ongoing. The study is expected to be completed by September 2021.

Preliminary results regarding real-time monitoring of emotional distress are available and will serve as a baseline for comparison of future versions of the AICF.

The PRIME framework has been configured to suit the above requirements and retrained using the CCC data and the human annotation of distress.

Using this data set and relevant literature, 2 clinical psychology doctoral students developed an annotation guideline (Multimedia Appendix 1). This guideline was used to categorize text-based data from one CCC online support group, comprising 8 participants over 10 sessions, by distress severity: low, moderate, moderate-high, or high level of distress, or “unable to identify” (Multimedia Appendix 2).

**Figure 3:**
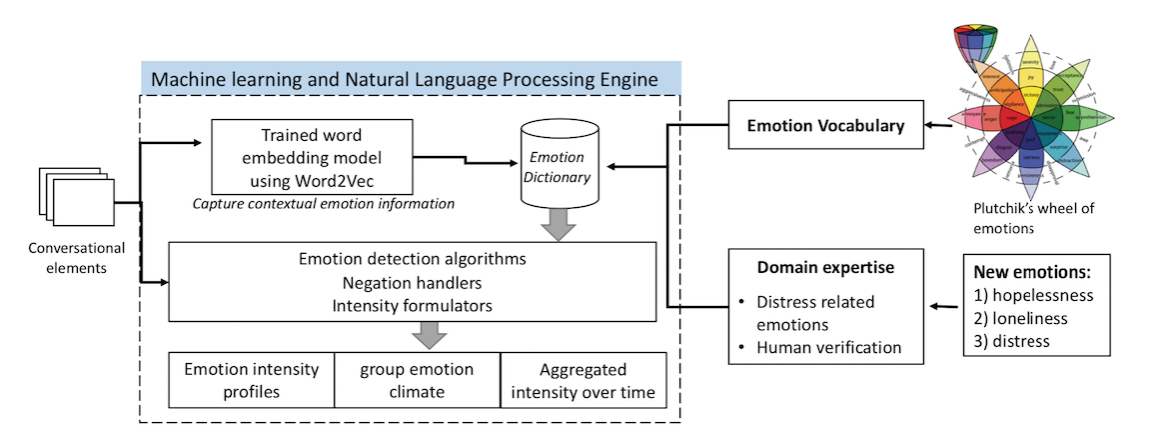
From PRIME to AICF prototype—3-stage transformation process.

The PRIME framework emotion detection algorithms were transformed for distress severity detection in the AICF in 3 stages (Figure 3). The outputs were used to visualize each participant and group emotion profiles, and monitoring emotion intensity fluctuations over time.

The intensity of each emotion for a given conversational extract is based on the output of the emotion classifier and the intensifier classifier. The emotion classifier detects each of the 11 emotions (8 basic emotions plus hopelessness, loneliness, and distress) while the intensifier classifier serves to augment or diminish the strength of each emotion. Table 3 presents several results from the initial experiment. The aggregate intensity for each emotion during the conversation is calculated using


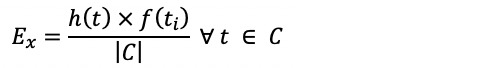


where *h* is the emotion classifier, *f* is the intensifier classifier, *t* is a word in set *C*, *t_i_* is the corresponding intensifier term, |*C*| is the count of words in *C*, and *E* is a vector of *x* emotions. This intensity score is designed to allow the AICF to track individual changes over time.

**Table 3:** Demonstration of emotion intensity calculation.

Extract	Emotion		Intensifier	
	Term	Classifier	Term	Classifier
“All the medicine I took make me too sleepy or sedated.”	—^a^	None	—	0.5
“yes *very exhausting*”	exhausting	sad	very	0.8
“This life is *very fragile*”	fragile	sad	very	0.8
“well I feel *totally useless* since I had this cancer”	useless	sad	totally	0.9
“I sense we are *losing energy*.”	losing energy	sad	—	0.5
“I feel *so depressed* regarding all this, but I went to see my family today”	depressed	hopelessness	so	0.7
“Relaxing days ahead no more treatments”	Relaxing	joy	—	0.5
“I love talking with this group!”	love	joy	—	0.5

^a^No term.

**Figure 4:**
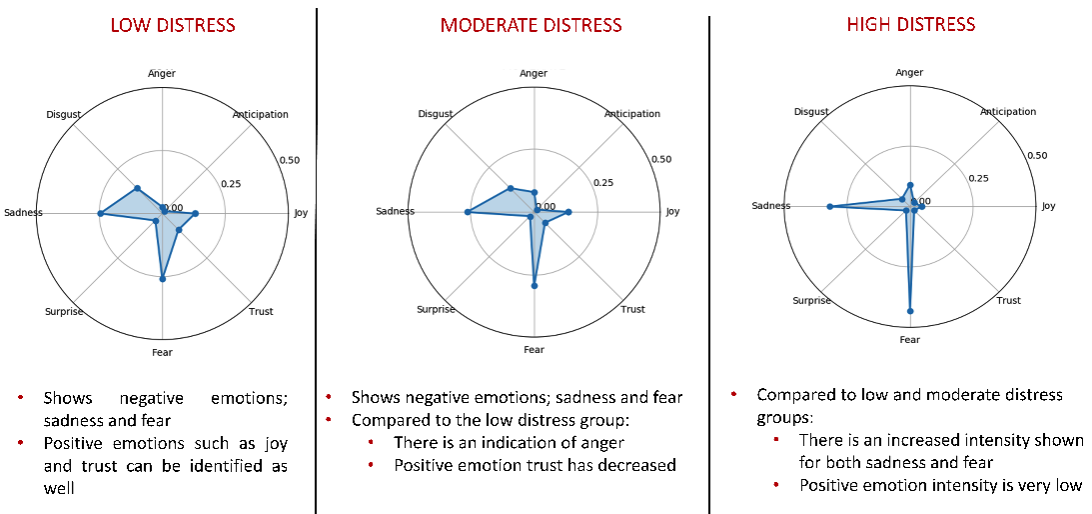
AICF classifications of emotions across levels of participant distress. Note: Given the small sample of data available, the emotion intensity scale was adjusted to 0.5.

Figure 4 shows radial graphs displaying the AICF classification of emotions expressed by individuals with different levels of distress. Distress was calculated as the aggregation of 4 negative basic emotions (anger, sadness, fear, disgust). According to the AICF, people with high distress experienced intense levels of sadness and fear, while those with moderate and low distress expressed similar profiles except that moderate distress individuals express more anger than their low distress counterparts.

When compared with the human annotation of distress levels, the AICF was able to correctly identify specific types and intensity of emotion from the 8 participants in 10 sessions for 78% of instances for low distress, 63% for moderate distress, and 85% for high distress. The baseline performance for distress classification was 72% agreement with the annotators with advanced training in clinical psychology.

## Discussion

### General

The AICF, an extension of the PRIME framework, represents a novel approach to help cancer online support group therapists track and monitor individual participant emotional text-based expressions that may indicate important mental health changes and outcomes, such as high distress, indicative of anxiety and depression. It also has the potential to track important online support group outcomes such as increased distress, participant engagement, and group cohesion.

Preliminary findings show that the AICF demonstrated acceptable performance in identifying emotional distress and target emotions. CCC patients experienced some distress when they signed up for online support groups which offered a rich emotional content of real-world data for distress detection algorithm training. Our results support the findings of Funk et al [53] who used a combination of techniques developed for basic emotion detections to predict clinically meaningful outcomes and symptoms of eating disorders, with a reported area under the receiver operating characteristic curve of 73%. However, the AICF has shown a lower accuracy (63%) in identifying moderate distress compared to the high (85%) and low distress (78%). One possible explanation is that moderate distress is a relatively ambiguous concept that even human experts may find it difficult to distinguish. The number of identified instances of moderate distress was lower than those of high and low distress as the human annotators were more likely to classify a distress statement as either high or low and these categories yielded the highest agreement between human annotators. Therefore, this contributed to the AICF having a hard time distinguishing the moderate level from the other levels of distress. The final evaluation will provide a more complete picture of distress identification.

The AICF has been designed to extract clinically important emotional outcomes based on a combination of basic emotions. While the literature predominantly focuses on sentiment or basic emotion detection on Twitter for commercial and political use [54-56], the AICF is better for clinical settings in several ways compared to previous automated deep emotion and intensity detection tools: The AICF was trained and applied on distress data from patients with cancer in clinical settings, instead of tweets that are free-flowing statements posted by the general public and that do not usually contain dialectic exchanges or symptoms or outcomes. Our data allow the AICF to identify more granular emotions and changes over time. The AICF has incorporated clinically trained therapists to identify distress and clinical outcomes from the data while most studies relied on hashtags to classify topics and emotions or on laymen annotators [55]. In one of the few studies [57] that attempted to identify emotions in a cancer population [57] using recurrent neural network models to extract common basic emotions such as fear and hope from tweets by patients with cancer, the authors identified joy as the most commonly shared emotion, followed by sadness and fear; these findings are similar to ours. However, the AICF will contribute to the uncharted areas of clinical psychology and psychiatry in which automatic emotion detection and classification systems have not yet been fully explored. This unique project attempts to identify complex emotions such as hopelessness and loneliness and that will open an avenue of research with clinical utilities.

The development phase was to evaluate the AICF performance on a training data set, to ensure that it is correctly classifying the key target outputs (detection of sadness and fear, engagement, group cohesion, emotional profiling in radar graphs). These features will be built into real-time and postgroup dashboard features for the online therapist leaders. The next phase involves human scoring for the emotion classification (eg, anger, joy) to ensure that the AICF is accurately labeling the emotion and its intensity. Once evaluated, this emotion data will be deployed in real-time engagement tracking and in the individual participant postsession summary, with end-user input on usability and effectiveness from the therapist leaders.

One limitation is that the AICF relies on human annotated training data and human scoring for performance evaluation. Human input can yield variable results as it requires judgement when classifying ambiguous posts. To reduce this variability, detailed literature-based guides were created for each emotion. Two doctoral-level clinical psychology–trained therapists created the guides and annotated the data independently, after which the results were reviewed. Another limitation is that the AICF prototype is based on PRIME, which was developed on data from patients with prostate cancer (all male participants, mostly aged 50 and older) whereas the CCC data represent a wider range of participant demographics and cancer types. A difference in distress and emotion expression is expected from the CCC participants in therapist-led synchronous groups. The AICF’s detection of more complex outcomes (eg, group cohesion) requires additional annotations, algorithm training, and performance evaluation, as this is a new broadly defined outcome, outside the basic emotions. Applications of any prototype such as the AICF require adaptations for the specific platform and desired outcomes. Lastly, due to protection of personal health information, the data deidentification process removed some of the information as the software has masked keywords that were also used in names.

### Implications

Online support groups are accessible and effective at reducing cancer-related emotional distress. However, therapists find it challenging to monitor individual participant distress and engagement in the absence of visual cues while responding to multiple participants’ messages simultaneously. Optimal online support group delivery and participant mental health outcomes require the therapist to effectively address and track markers of high distress. These markers include participants posting messages indicating intense sadness or fear, or participants withdrawing due to emotional dysregulation. An AICF can assist by detecting and flagging issues (eg, a spike of distress) that are amenable to treatment in real time, thus allowing therapists to provide higher levels of individual support. An AICF presents a unique opportunity to strengthen person-centered care in the online support group settings by attending to individual needs while expanding access to high-quality virtual health care.

## Appendix

Multimedia Appendix 1 Distress human annotation guidelines.

Multimedia Appendix 2 Distress annotation method.

Multimedia Appendix 3 OICR-CCO Health Services Research Grant peer review reports.

## Abbreviations

AI: artificial intelligence
AICF: artificial intelligence–based cofacilitator
CCC: Cancer Chat Canada
HADS: Hospital Anxiety and Depression Scale
NLP: natural language processing
PRIME: Patient-Reported Information Multidimensional Exploration
