# Supplementary material for: Natural Language Processing–Based Virtual Cofacilitator for Online Cancer Support Groups: Protocol for an Algorithm Development and Validation Study
Source: JMIR Res Protoc. 2021 Jan 7;10(1):e21453. doi: 10.2196/21453 (PMC7819785; doi:10.2196/21453)
Supplement: Multimedia Appendix 1 [file resprot_v10i1e21453_app1.docx]

**Annotation Guide for Distress**

**1. DISTRESS**

a.) HIGH
 b.) MODERATE-HIGH
 c.) MODERATE
 d.) LOW

**2. NON-DISTRESS**

a.) GENERAL NON-DISTRESS
 b.) POSITIVE STATEMENTS

**3. UNABLE TO CODE**

**4. UNSURE**

**Criteria for Annotation – Description of Codes**

**1. DISTRESS**

**a.) HIGH** includes
 - Anhedonia
 - Complaints of significant pain
 - Fear or great anxiety
 - Helplessness
 - Hopelessness or giving up
 - Lack of energy
 - Shock
 - Persistent thoughts about death/mortality
 - Any of the below expressed as ‘severe, serious, constant’.

e.g. “I want to learn to deal with the enormous anxiety of having cancer.”
e.g. “I worry more about the ones that I will leave behind.”

**b.) MODERATE-HIGH** includes
 - Isolation
 - Fear or great anxiety but trying to cope
 - Helplessness but trying to cope

e.g “coping with fear and anxiety is my biggest challenge”

e.g. “I am not seeing a counsellor. I know I need to see one but I am trying to keep it to myself. I know it’s not good.”

**c.) MODERATE** includes
- Anger/annoyance
- Anxious but trying to overcome
- Confusion
- Frustration
- Guilt
- Helplessness but trying to overcome
- Some physical symptoms but trying to cope

e.g. “I broke my arm about a month ago so I am not sure how long I can type before it hurts too much”
e.g. “anxiety is a huge problem for me too, I try to keep busy – reading, watching TV, walking, listening to music, playing computer games etc. I have been on Cipralex for years and it helps me cope better overnight.”

**d.) LOW** includes
- Acceptance of distress
- Coping well with challenges
- Mild anxiety symptoms
- Disappointment (mild)
- Mild or fairly well-managed physical symptoms
- Mild confusion
- Uncertainty

e.g. “I was prescribed sleeping pills, I forgot the name but I tossed them as I was up all night” e.g. “I think of my cancer as an unwelcome guest and I do everything possible to keep it from getting comfortable.”

**2. NON-DISTRESS**

**a.) GENERAL NON DISTRESS** includes any full-sentence statements which are neutral and have little to no ‘negative’ emotional weight

e.g. “I am trying to get this to work on my iPad but it only works from my laptop”
e.g. “Have you been giving yourself Neupogen shots?”

**b.) POSITIVE STATEMENTS
 -** Clearly hopeful
 **-** Encouraging
 **-** Enthusiastic
 **-** Optimism
 **-** Supportive

e.g. “I love talking to survivors. They have a sparkly in their eyes. It is like you know that they survived something bad but came out after that”
e.g. “I am amazed how fast our group has become connected”

**3. UNABLE TO CODE
-** short (under 5 words), incomplete sentences
**-** nonsensical or mostly using smileys/emoticons

e.g. lol
e.g. hey guys, great <smiley><heart> etc.
