# Supplementary material for: Natural Language Processing–Based Virtual Cofacilitator for Online Cancer Support Groups: Protocol for an Algorithm Development and Validation Study
Source: JMIR Res Protoc. 2021 Jan 7;10(1):e21453. doi: 10.2196/21453 (PMC7819785; doi:10.2196/21453)
Supplement: Multimedia Appendix 2 [file resprot_v10i1e21453_app2.docx]

**Distress Annotation**

- 1. Two PhD students in Clinical and Counselling Psychology were recruited from the University of Toronto (the “**Annotators**”). The transcripts were from a Cancer Chat online support group of Active Treatment participants which took place Winter 2018/2019. They were given the same transcript set comprising all eight sessions for this particular group.
  2. The Annotators separately reviewed the complete, eight session transcript set. They annotated all statements in the transcript set using “nodes” in Nvivo 12 according to the framework mentioned in APPENDIX A – Annotation Guidelines. This guideline was given and explained to both Annotators prior to the beginning of their work on the transcript.
  3. Statements which were ambiguous were annotated as UNSURE and were later reviewed by both Annotators during their first consensus meeting on August 23, 2019.
  4. The two annotated files were merged together and a coding comparison was run in Nvivo 12 to check their agreement. It is noteworthy that inter-rater agreement in Nvivo 12 is not to be relied on too heavily, rather a team-based consensus method is preferred for research with qualitative data. It was run only as a preliminary check to highlight differences and refine the annotation guidelines.
  5. The Annotators ran a preliminary test of inter-rater reliability for the DISTRESS, NON-DISTRESS and POSITIVE STATEMENTS, which yielded results of over 90% agreement on these target groupings. The lowest agreement was just over 60% for the groupings of UNABLE TO CODE and GENERAL NON-DISTRESS, which led the Annotators to revise their annotation guidelines. It was felt this difference was due to a lack of clarity about which statements were too short or irrelevant to code. This was revised and amended in creating the final consensus document.
  6. The Annotators traded their versions of the annotated transcript set and reviewed each other’s work, taking detailed notes of where and why they labeled some statements differently.
  7. Once transcripts were reviewed separately, the two Annotators reviewed all differences in annotated statements together and came to a consensus for each statement. This process is detailed in the file “Team Annotation Review.nvp” which includes notes on each statement for which the Annotators disagreed. Most of the differences were regarding the levels at which to code distress (high, moderate-high, moderate or low). None of the discrepancies were more than one level apart (e.g. one rater coded as ‘moderate’ while another coded the same sentence as ‘moderate-high’).
  8. An integrated file was created which reflected the consensus product of the two Annotators’ agreement. This file was reviewed together one final time by both Annotators to ensure its accuracy.
