# Supplementary material for: Natural Language Processing–Based Virtual Cofacilitator for Online Cancer Support Groups: Protocol for an Algorithm Development and Validation Study
Source: JMIR Res Protoc. 2021 Jan 7;10(1):e21453. doi: 10.2196/21453 (PMC7819785; doi:10.2196/21453)
Supplement: Multimedia Appendix 3 [file resprot_v10i1e21453_app3.pdf]

Reviewer Report
HSR Full Application

1. Applicant information:	
**Applicant ID**	P.HSR.147
**Project Title**	Building an Artificial Intelligent System to Enhance Online Support Group in Cancer
**PI(s) and institution(s)**	Mary Jane Esplen, UofT; Yvonne Leung, De Souza Institute, UHN

2. **Reviewer information:** This information will not be disclosed to the candidate.	
**Reviewer type**	Primary reviewer

3. Brief summary of the proposal: This has been copied and pasted from review of the Letter of Intent – edit as appropriate. The purpose of this summary is so you can easily recall the proposal should it be discussed during the panel meeting.

Cancer Chat Canada (CCC) is a therapist led text-based online support group (OSG) service, which is designed to address cancer-related distress. Every OSG is formed with one therapist and 6-8 participants, meeting weekly for 8 weeks in a private forum (chatroom). OSG vary in their health-related goals, but they generally provide online care to support adjustment to patients or their caregivers and to address educational needs and specific concerns (e.g. sexual functioning; bereavement). However, therapists often feel challenged to lead OSG while addressing individual group member's distress / needs in the absence of visual cues.

AI can analyse medical notes to help detect the risk of mental health problems, automatically recommend resources, and monitor moods. Investigators propose to develop and evaluate an 'Artificial Intelligent Based Co-facilitator (AICF)' to track and monitor OSG participants' distress through a real-time analysis of texts posted during the sessions.

Specifically, AICF will: 1. generate participant profiles with discussion topic summaries and emotion trajectories for each session, 2. identify participant(s) at-risk for increased emotional distress and alert the therapist for follow-up, and 3. automatically suggest tailored recommendations based on participant needs.

AICF allows real time detection of issues that are amenable to treatment, thus allowing therapists to be more proactively support each group member. This project has direct relevance in improving online psychosocial oncology services currently being delivered by CCC to support Canadians across Canada including the 14 regional cancer centres in Ontario.

Work will build upon existing 'Patient-Reported Information Multidimensional Exploration (PRIME)' framework, an automated ensemble of deep-learning based natural language processing techniques was developed to analyze texts from online patient forums. PRIME has been successfully applied to ten, high-volume, online patient forums consisting of 22,233 prostate cancer patients, which generated a text dataset of 609,960 conversations. PRIME demonstrated its capabilities in identifying diverse physical symptoms, and functional and emotional outcomes (e.g. sadness, anger, confusion) embedded in forum discussions.
Four phases:

1) Develop AICF, and via data-based algorithms, “learn” to generate patient profiles, provide monitoring and alerts, and recommend tailored resources.
2) Validate AICF by testing it in an entirely different dataset.
3) Beta-test AICF within CCC (in real time)
4) Evaluate user experiences

4. Evaluation: Provide written comments for each criterion listed below. The text of this report will be provided to the applicant verbatim and as such, no reviewer identifier should be used.

Potential to provide a positive impact for Ontarians (e.g., address an unmet clinical need, improve patient outcomes and population health and/or improve the health care system)

Online support groups represent an important tool for patients. CCC has had a proven positive impact for patients so the proposed work aims to further enhance the value of this forum to more patients. Proposed work, if operationalized successfully, could result in early detection of concerning distress and also lead to more tailored recommendations.

Originality and innovativeness of the approach (e.g., advances new methods, uses existing methods to generate new empirical evidence, etc.)

Work seems to be mainly based on existing PRIME framework in prostate cancer so not 100% novel per se, but investigators propose to adapt this into CCC.

Feasibility of the research plan and strength of the research team.

Investigators have the skillset, but the 4 phases of work seem rather ambitious to conduct.

5. Additional comments

Appropriateness of the budget

Seems appropriate; mainly personnel.

Other

N/A

6. Scores: Referring to Table 1 below, provide a score for each criterion and the project overall. *The overall project score is not a sum nor an average score of each criterion*. It is a separate score assigned to the overall proposal. Reviewers are encouraged to utilize the full range of the scoring scale to reduce the clustering of applications.

Potential to provide a positive impact for Ontarians	4.6
Originality and innovativeness of the approach	4.0
Feasibility of the research plan and strength of the research team.	3.2
**Overall project score**	3.8

Table 1: Scoring guide for each criterion and the project overall.

Score	Description
4.7 – 5.0	Excellent with no weaknesses identified
4.2 – 4.6	Excellent with minor weaknesses identified
3.6 – 4.1	Very good with minor weaknesses identified
3.0 – 3.5	Very good with moderate weaknesses identified
2.4 – 2.9	Good with moderate weaknesses identified
1.7 – 2.3	Fair with moderate weaknesses identified
1.0 – 1.6	Poor with moderate to major weaknesses identified
Below 1.0	Poor with major weaknesses identified

Reviewer Report
HSR Full Application

**1. Applicant information:**	
**Applicant ID**	P.HSR.147
**Project Title**	Building an Artificial Intelligent System to Enhance Online Support Group in Cancer
**PI(s) and institution(s)**	Mary Jane Esplen, UofT; Yvonne Leung, De Souza Institute, UHN

**2. Reviewer information:** This information will not be disclosed to the candidate.	
**Reviewer type**	Secondary reviewer

3. Brief summary of the proposal: This has been copied and pasted from review of the Letter of Intent – edit as appropriate. The purpose of this summary is so you can easily recall the proposal should it be discussed during the panel meeting.

This is an interesting proposal to utilize AI as a means of assisting with the identification of "at risk" participants of an online support group (CCC). The proposal aims to build on similar technology being used in Australia to develop, validate and test the utility of this process in cancer patients.

4. Evaluation: Provide written comments for each criterion listed below. The text of this report will be provided to the applicant verbatim and as such, no reviewer identifier should be used.

Potential to provide a positive impact for Ontarians (e.g., address an unmet clinical need, improve patient outcomes and population health and/or improve the health care system)

Given the limited availability of psychological support for cancer patients and the established online forum of group support this seems like a potentially valuable way of increasing the reach of psychologists providing this care.

Originality and innovativeness of the approach (e.g., advances new methods, uses existing methods to generate new empirical evidence, etc.)

Both original and innovative

Feasibility of the research plan and strength of the research team.

It appears that the research plan is feasible and that the team has the breadth and expertise to complete the proposed work

5. Additional comments

Appropriateness of the budget

No concerns

Other

My main concern is the ability of machine learning to adapt to different cultural subgroups. The applicants do not address this and I personally do not know this field well enough to know if this would be an issue or not

**6. Scores:** Referring to Table 1 below, provide a score for each criterion and the project overall. *The overall project score is not a sum nor an average score of each criterion*. It is a separate score assigned to the overall proposal. Reviewers are encouraged to utilize the full range of the scoring scale to reduce the clustering of applications.	
Potential to provide a positive impact for Ontarians	4.6
Originality and innovativeness of the approach	4.7
Feasibility of the research plan and strength of the research team.	4.7
**Overall project score**	4.7

Table 1: Scoring guide for each criterion and the project overall.

Score	Description
4.7 – 5.0	Excellent with no weaknesses identified
4.2 – 4.6	Excellent with minor weaknesses identified
3.6 – 4.1	Very good with minor weaknesses identified
3.0 – 3.5	Very good with moderate weaknesses identified
2.4 – 2.9	Good with moderate weaknesses identified
1.7 – 2.3	Fair with moderate weaknesses identified
1.0 – 1.6	Poor with moderate to major weaknesses identified
Below 1.0	Poor with major weaknesses identified
